# Neutrophils in triple-negative breast cancer: an underestimated player with increasingly recognized importance

**DOI:** 10.1186/s13058-023-01676-7

**Published:** 2023-07-26

**Authors:** Chanjuan Zheng, Xi Xu, Muyao Wu, Lian Xue, Jianyu Zhu, Hongzhuo Xia, Siyu Ding, Shujun Fu, Xinyu Wang, Yian Wang, Guangchun He, Xia Liu, Xiyun Deng

**Affiliations:** 1 Key Laboratory of Translational Cancer Stem Cell Research, Department of Pathophysiology, Hunan Normal University School of Medicine, Changsha, Hunan China; 2 Department of Biochemistry and Molecular Biology, Jishou University, Jishou, Hunan China; 3 Key Laboratory of Study and Discovery of Small Targeted Molecules of Hunan Province, Hunan Normal University School of Medicine, Changsha, Hunan China; 4 Department of Toxicology and Cancer Biology, University of Kentucky, Lexington, KY USA

**Keywords:** Neutrophil, Triple-negative breast cancer, Tumor microenvironment, Metastasis, Immunotherapy

## Abstract

Triple-negative breast cancer (TNBC) is the most lethal subtype of breast cancer, with limited therapeutic options readily available. Immunotherapy such as immune checkpoint inhibition has been investigated in TNBC but still encounters low overall response. Neutrophils, the most abundant leukocytes in the body, are increasingly recognized as an active cancer-modulating entity. In the bloodstream, neutrophils escort circulating tumor cells to promote their survival and stimulate their proliferation and metastasis. In the tumor microenvironment, neutrophils modulate the immune milieu through polarization between the anti-tumor and the pro-tumor phenotypes. Through a comprehensive review of recently published literature, it is evident that neutrophils are an important player in TNBC immunobiology and can be used as an important prognostic marker of TNBC. Particularly, in their pro-tumor form, neutrophils facilitate TNBC metastasis through formation of neutrophil extracellular traps and the pre-metastatic niche. These findings will help advance the potential utilization of neutrophils as a therapeutic target in TNBC.

## Background

Neutrophils, also known as polymorphonuclear cells, are a population of granulated cells of the myeloid lineage. They are the most abundant leukocytes in the bloodstream, constituting 50–70% of all circulating leukocytes in adult humans [1]. Although neutrophils primarily play a role in bacterial infections [2], there is increasing evidence that neutrophils play an important role in cancer initiation and progression [3]. Neutrophils are actively involved in various aspects of breast cancer development, including growth, migration/invasion, angiogenesis, and metastasis [4–6].

Breast cancer has become the most commonly diagnosed malignancy worldwide [7]. Triple-negative breast cancer (TNBC), defined by the lack of expression of estrogen receptor (ER), progesterone receptor (PR), and absence of overexpression/amplification of human epidermal growth factor receptor 2 (HER2), is the most aggressive and difficult-to-treat subtype of breast cancer [8, 9]. Although immunotherapies are effective in some patients, the majority of TNBC cases still do not benefit from immunotherapies. Recently, the identification of neutrophils as an active contributing factor and a novel therapeutic target in cancer has shed light on and has shown great promise for breast cancer therapy. This review presents the most recent developments concerning the involvement of neutrophils in TNBC.

### Roles of neutrophils in TNBC

Over the past decade, tumor-associated neutrophils (TANs) have been increasingly recognized as a key functional identity with the potential to impact breast cancer prognosis, especially the TNBC subtype [10–12]. TANs can exist in the circulation (circulating neutrophils) as well as in the microenvironment (tumor-infiltrating neutrophils).

### Circulating neutrophils in TNBC

Circulating tumor cells (CTCs) enter the vasculature from the primary tumor site and contain metastasis-initiating cells that are able to extravasate and colonize secondary organs [13]. It is now clear that circulating immune cells can form clusters with CTCs and help them survive the harsh hemodynamic conditions and metastasize. Neutrophils could attach to CTCs, which leads to the adhesion of tumor cells to the blood vessels, ultimately leading to transendothelial migration and extracellular matrix remodeling [14]. In a 2014 report, clustered CTCs were found to be more competent than individual circulating breast cancer cells at surviving and metastasizing [15]. To investigate the type of leukocytes in determining the fate of CTCs, the same group analyzed blood samples from 70 breast cancer patients and 5 breast cancer-bearing mouse models. They found that the majority (75% in human patients and 80.5–91.7% in mice) of leukocytes that escort circulating breast cancer cells are neutrophils. When neutrophils are depleted in breast cancer-bearing mice, the formation of lung metastasis is delayed. In addition, injection of CTCs isolated from the CTC-neutrophil clusters into the circulation gives rise to a considerably higher number of metastases to the lung than unclustered CTCs [16].

**Fig. 1:**
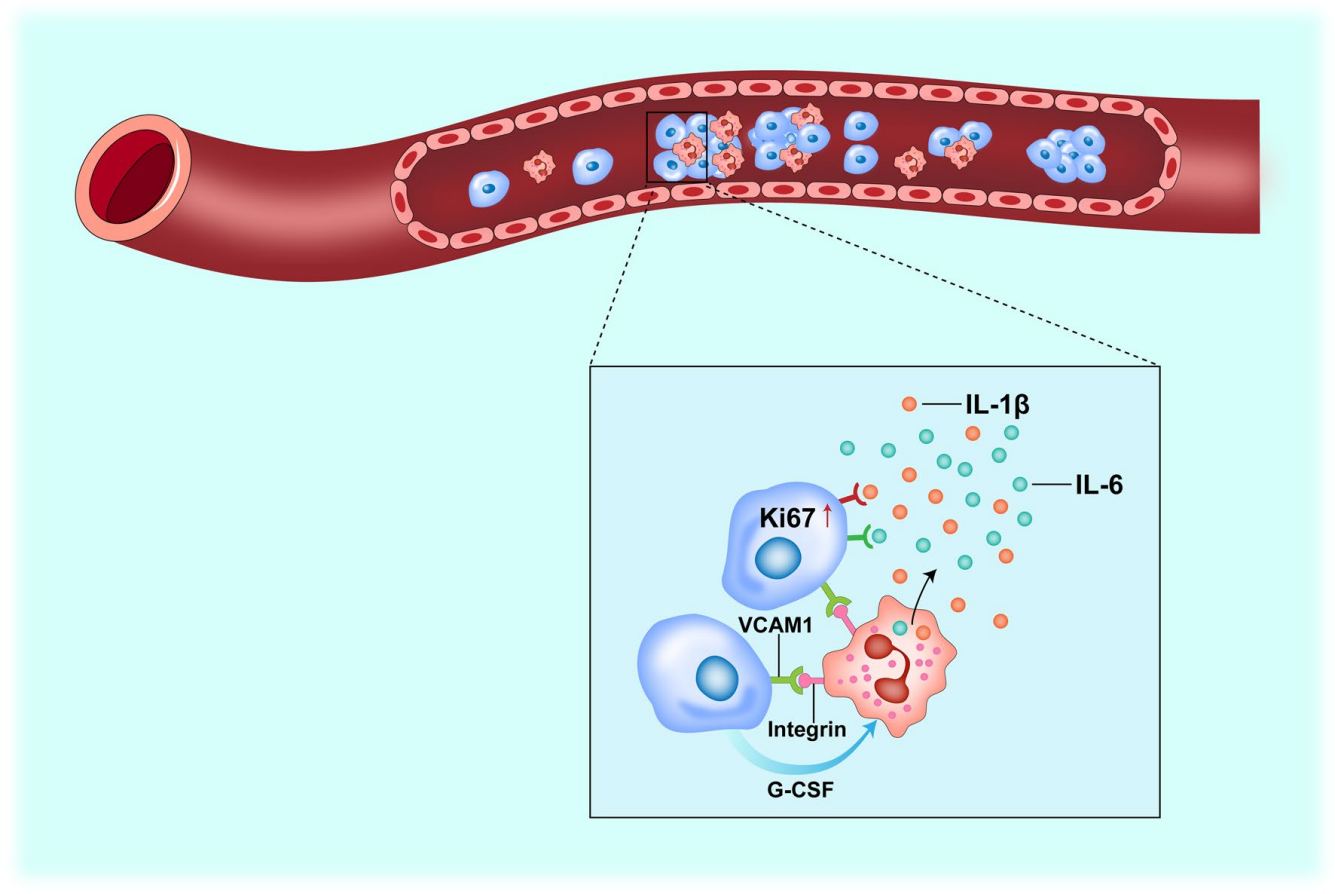
Neutrophils help CTCs survive, proliferate, and metastasize. Interactions with neutrophils enhance the survival, proliferation, and metastasis of circulating tumor cells (CTCs) in the bloodstream. Expression of vascular cell adhesion molecule 1 (VCAM1) in cancer cells mediates the formation of CTC–neutrophil clusters, through binding to specific integrins such as α3β1 integrin on neutrophils. CTCs also express G-CSF to stimulate the pro-tumor functions of neutrophils. In turn, neutrophils clustered with CTCs secrete cytokines such as IL-1β and IL-6, which promote CTC survival and proliferation through increased expression of Ki67

Why do CTCs that are associated with neutrophils metastasize more readily than those that are not? Several mechanisms account for the enhanced ability of CTCs clustered with neutrophils. First, in the neutrophil–CTC cluster, neutrophil-released cytokines such as IL-6 and IL-1β render CTCs more competent in cell cycle progression through a higher expression of proliferation-related genes such as Ki67 [16]. Second, granulocyte colony-stimulating factor (G-CSF) secreted by cancer cells stimulates the pro-tumor function of neutrophils in invasive breast cancer [17] (Fig. 1). Breast cancer patients, including TNBC patients, having CTC–neutrophil clusters show worse progression-free survival than those having unclustered CTCs. Circulating neutrophils endow CTCs with higher malignant potency [16].

### Tumor-infiltrating neutrophils in TNBC

Tumor-infiltrating neutrophils (TINs) are the neutrophils that interact with cancer cells in the tumor microenvironment (TME) [11, 18]. Wang et al. demonstrated that high levels of TINs are associated with advanced histologic grade and tumor stage as well as the TNBC subtype. Mechanistically, TINs induce migration, invasion, and epithelial-to-mesenchymal transition (EMT) of breast cancer cells through production of tissue inhibitor of metalloproteinase 1 (TIMP1) and subsequent induction of CD90 [19].

**Fig. 2:**
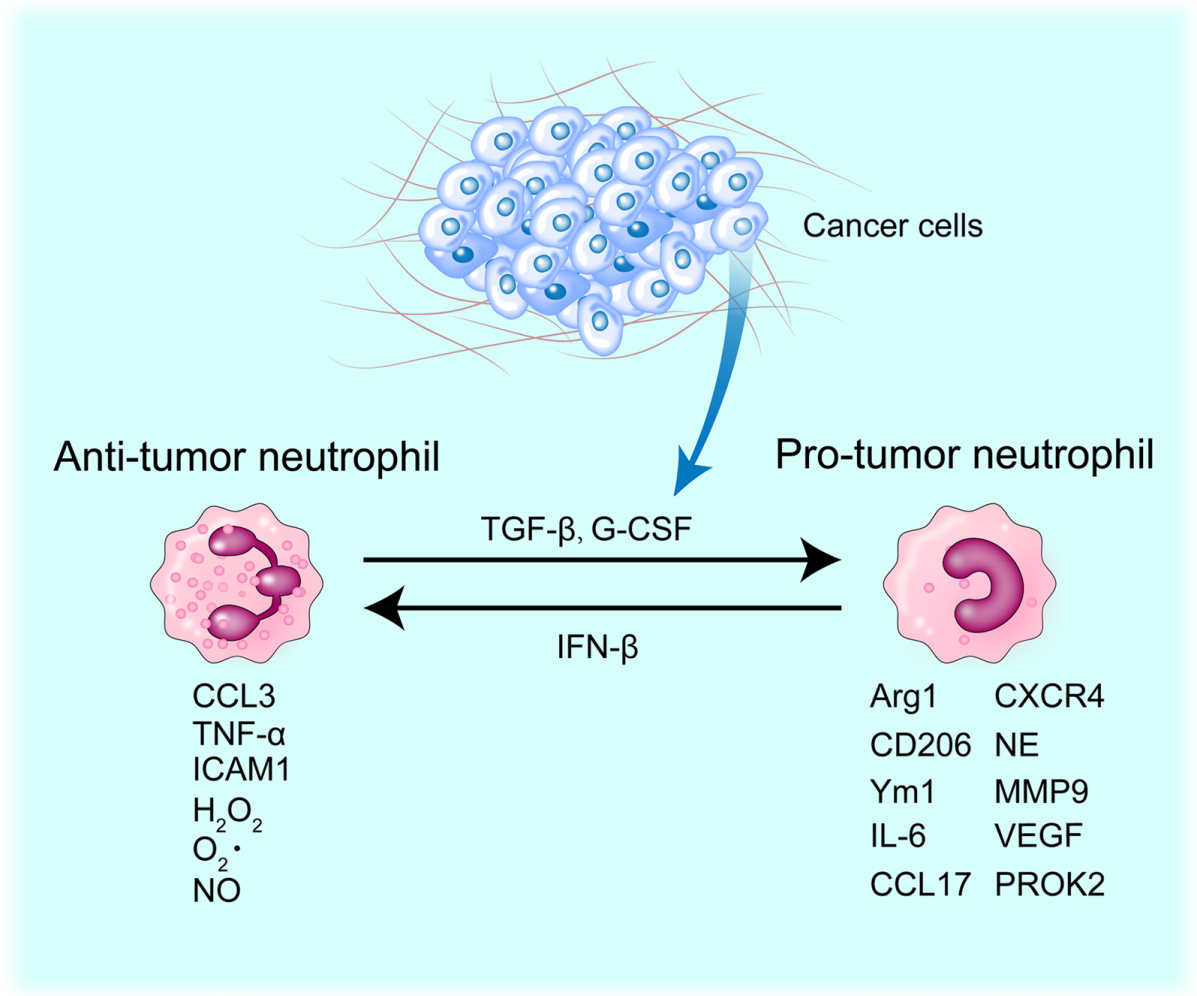
Neutrophil reprogramming in the tumor microenvironment. During cancer progression, cytokines, particularly TGF-β and G-CSF, secreted from tumors (and probably stromal cells) stimulate transition of neutrophils from an anti-tumor to a pro-tumor phenotype. Conversely, pro-tumor neutrophils can be reprogrammed by cytokines (i.e., IFN-β), released from the tumor microenvironment such as dendritic cells and/or macrophages to become anti-tumorigenic. Basically, the anti-tumor neutrophils are developmentally more mature with a more segmented nucleus than the pro-tumor cells, which have a more ring-like structure of the nucleus. The anti-tumor neutrophils express higher levels of cytokines such as CCL3, TNF-α, and ICAM1. They also have higher levels of ROS such as H_2_O_2_ and O_2_‧, and RNS such as NO. In contrast, the pro-tumor neutrophils have higher levels of Arg1, CD206, Ym1, IL-6, CCL17, CXCR4, NE, MMP9, VEGF, and PROK2

TINs can play quite different functions in the TME through a process called polarization. Polarization of neutrophils was first reported in 2009 by Fridlender and colleagues, who proposed the name N1 (anti-tumor) versus N2 (pro-tumor) to describe a process of morphological and functional reprogramming of TANs in a similar way to M1 versus M2 tumor-associated macrophages (TAMs) [20]. However, recent studies have revealed the complexity of neutrophil subpopulations and their functions in the TME, making it difficult to simply classify them as N1 or N2. For the purpose of generalization and clarity in this review, neutrophils are divided into the anti-tumor and pro-tumor types. Three tumor-derived cytokines, i.e., transforming growth factor-β (TGF-β), G-CSF, and interferon-β (IFN-β), are the most studied molecules involved in polarization of neutrophils. While TGF-β and G-CSF activate a tumor-promoting program of neutrophils, i.e., pro-tumor polarization [21], IFN-β promotes the reverse process, i.e., anti-tumor polarization [22] (Fig. 2). In TNBC, tumor cell-derived conditioned medium induces a polarized morphology and activation of neutrophils, mediated by cytokines such as TGF-β and the ligands for the chemokine receptor CXCR2, i.e., CXCL1/2/3 [23, 24]. In addition to these well-known cytokines, studies in other cancer types have shown that tumor can regulate neutrophil polarization through other factors. For example, IFN-γ combined with GM-CSF also drives neutrophils toward an anti-tumor state [25]. Whether these factors also contribute to neutrophil polarization in TNBC needs to be validated.

The anti-tumor TANs have higher levels of the C–C motif chemokine CCL3, tumor necrosis factor-α (TNF-α), and intercellular adhesion molecule 1 (ICAM1). The anti-tumor TANs also have higher levels of reactive oxygen species (ROS) such as hydrogen peroxide (H_2_O_2_) and superoxide anion (O_2_‧) as well as reactive nitrogen species (RNS) such as nitric oxide (NO). In contrast, the pro-tumor TANs have higher levels of Arginase 1 (Arg1), CD206, Ym1, IL-6, CCL17, CXCR4, neutrophil elastase (NE), matrix metalloproteinase 9 (MMP9), vascular endothelial growth factor (VEGF), and the neuropeptide prokineticin 2 (PROK2, also known as Bv8) [4, 20, 26, 27]. Although neutrophils in their pro-tumor form also produce ROS, the level of ROS is relatively low (Fig. 2). It should be noted that the separation of the neutrophil functions as being anti-tumor or pro-tumor is probably oversimplified. Moreover, it is assumed that the differential roles of neutrophils may depend on the different stages of cancer development, with an anti-tumor role at a relatively early stage and a pro-tumor role at late stages of carcinogenesis [1]. However, this assumption needs to be further validated using multiple experimental systems.

### Neutrophil extracellular traps in TNBC

The neutrophil extracellular trap (NET) is a peculiar web-like structure containing DNA strands in a complex with histones and neutrophil-derived enzymes such as NE, MMP9, myeloperoxidase (MPO), and peptidylarginine deiminase 4 (PAD4) [28, 29]. Neutrophils contribute to metastasis of a variety of tumors through the formation of NET. In mouse metastatic seeding models, formation of NETs has been found to be an important function of neutrophils that promotes breast cancer liver [30] and lung [31] metastasis. Notably, the number of NETs varies across different breast cancer subtypes, with the highest number observed in TNBC [31].

**Fig. 3:**
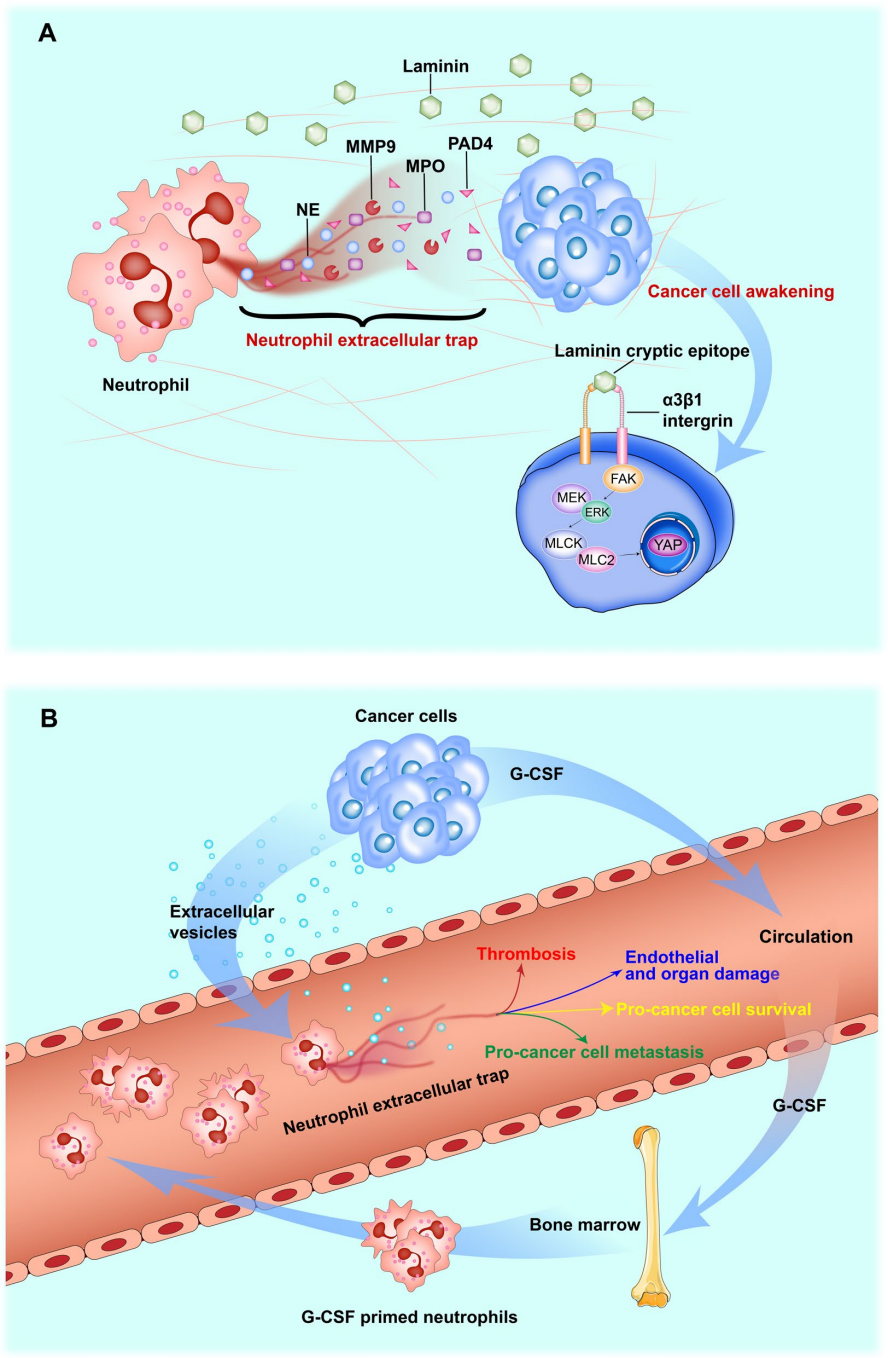
Roles of NETs in promoting malignant progression and awakening dormant cancer cells. The neutrophil extracellular trap (NET) is composed of neutrophil-derived DNA strands and enzymes such as NE and MMP9. NETs exist in the circulation as well as in the tumor microenvironment. **A** In the circulation, G-CSF derived from cancer cells acts in an endocrine manner to stimulate mobilization of neutrophils from the bone marrow to the bloodstream, leading to neutrophilia. In addition, tumor-released EVs (including exosomes) interact with the circulating neutrophils favoring the formation of NETs. NETs in the circulation promote cancer progression through several interrelated ways: (1) stimulating the formation of cancer-associated thrombosis; (2) promoting endothelial damage and organ dysfunction; (3) facilitating cancer cell survival; and (4) promoting cancer cell metastasis. In the tumor microenvironment, NE and MMP9 contained in NETs cleave laminin in the extracellular matrix, resulting in the exposure of cryptic epitopes on laminin. Exposed laminin epitopes trigger the proliferation of cancer cells through activation of the α3β1 integrin-mediated signaling pathway, leading to the awakening of dormant cancer cells

In the circulation, NETs can be formed by two cancer cell-related mechanisms. First, increased mobilization of neutrophils from the bone marrow, stimulated by tumor cell-secreted G-CSF, results in an increased count of circulating neutrophils called neutrophilia. Second, the factors carried in tumor cell-derived extracellular vesicles (EVs) stimulate the formation of NETs in the circulation (Fig. 3A). In turn, circulating NETs favor cancer progression through (1) stimulating the formation of cancer-associated thrombosis; (2) promoting endothelial damage and organ dysfunction; (3) facilitating cancer cell survival; and (4) promoting cancer cell metastasis [32, 33].

In the TME, NETs can awaken breast cancer cells by helping them exit the dormancy program (Fig. 3B). In doing this, the proteolytic enzymes NE and MMP9 contained in the NET cleave laminin in the extracellular matrix, resulting in the exposure of a cryptic epitope within laminin. In turn, dormant cancer cells trapped by NETs can sense the newly formed laminin epitopes through an α3β1 integrin-mediated mechanism, leading to activation of the proliferative program and, thus, awakening dormant breast cancer cells [34].

### Neutrophils and formation of the pre-metastatic niche in TNBC

Since the first proposal by Kaplan et al. [35], the concept of the “pre-metastatic niche” has been well accepted and demonstrated in several types of cancer, including TNBC. A pre-metastatic niche can be defined as a supportive and permissive microenvironment within a target organ undergoing a series of cancer cell-induced molecular and cellular changes favoring colonization and growth of cancer cells before their arrival [36, 37]. The characteristic features of the pre-metastatic niche involved in TNBC metastasis include: (1) inflammation as manifested by recruitment of bone marrow-derived cells, including neutrophils; (2) immunosuppression; (3) increased vascular permeability and angiogenesis; and (4) extracellular matrix remodeling [37–39].

In TNBC, tumor cells could regulate neutrophils by releasing EVs to facilitate pre-niche formation. miR-200b-3p derived from TNBC exosomes induces the upregulated expression of CCL2 by alveolar epithelial type II cells, and these chemokine ligands recruit neutrophils and promote pre-metastatic niche formation in the lung [40]. Lin28B, a conserved RNA-binding protein, is particularly upregulated in the TNBC subtype. In mouse models of TNBC, tumor-derived EVs containing the pluripotent factor Lin28B were found to upregulate the expression of CXCLs (CXCL1, CXCL2, CXCL3, and CXCL5) in the lung tissue, resulting in neutrophil infiltration. Lin28B increased IL-6 and IL-10 production, which could convert infiltrated neutrophils from tumor-suppressive to tumor-promoting. These pro-tumor neutrophils can inhibit T cell growth and activation and Th1 cell differentiation through various mechanisms. IL-10 can stimulate pro-tumor neutrophils to produce PD-L2, which inhibits T cells, while IL-6 can enhance this process. Additionally, pro-tumor neutrophils can inhibit IL-12, a crucial factor in Th1 cell differentiation [41].

**Fig. 4:**
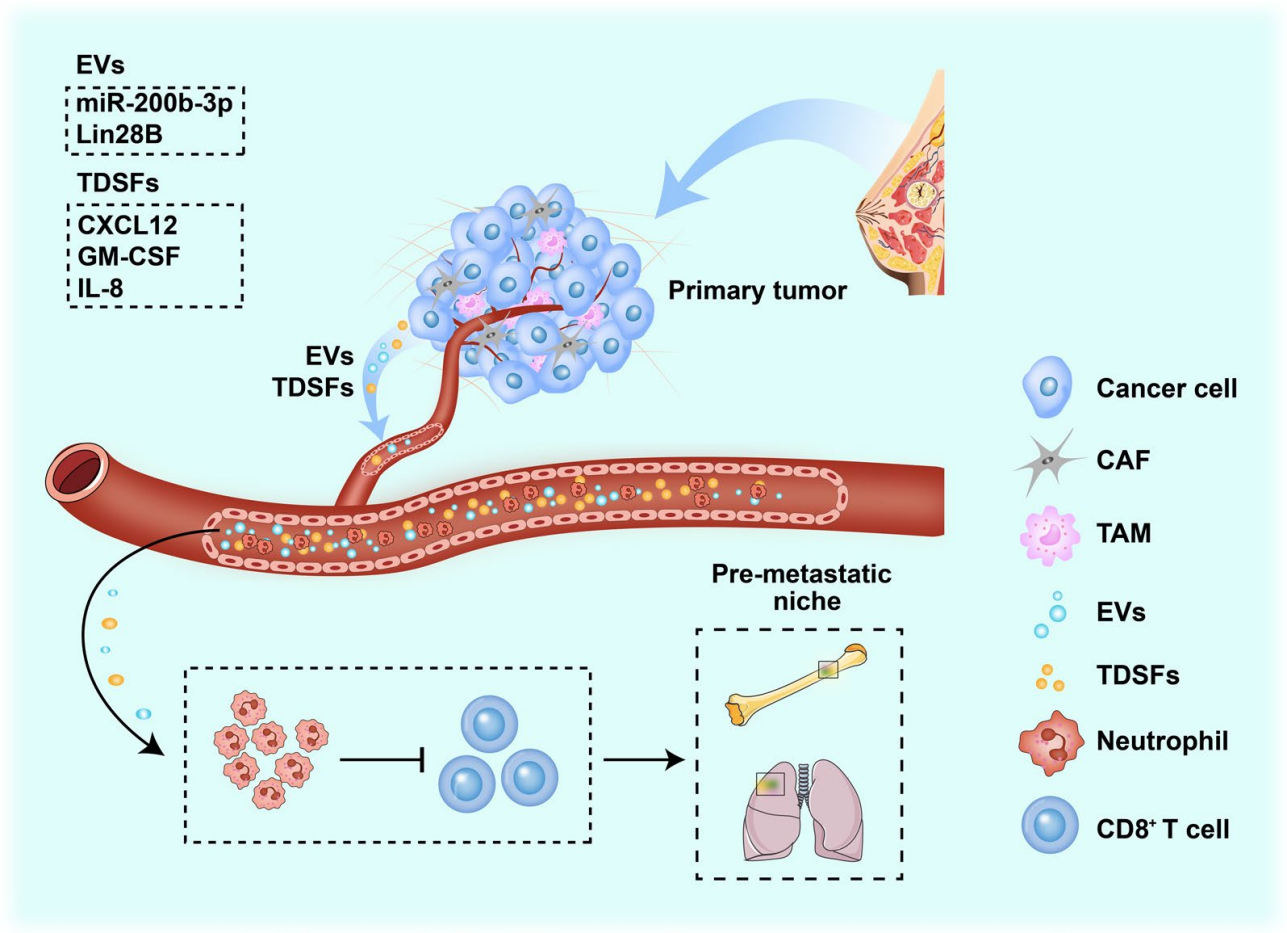
Tumor cell-induced formation of the pre-metastatic niche. Tumor-derived EVs (miR-200b-3p, Lin28B) and tumor-derived secreted factors (TDSFs) (CXCL12, GM-CSF, IL-8) enter the metastatic target organ through the circulation. These factors produce an inhibitory effect on cytotoxic T lymphocytes mediated by tumor-infiltrating neutrophils, leading to the formation of a receptive microenvironment in the target organ including the bone and the lung for the cancer cells prior to their arrival

In addition to EVs, tumor-derived secreted factors (TDSFs) play a critical role in the formation of the pre-metastatic niche. TDSFs include IL-6, IL-8, IL-1β, G-CSF, GM-CSF, and chemokines such as CCL2, CXCL12/SDF-1, and CXCL1 [42]. G-CSF derived from TNBC cells recruiting Ly6G^+^ Ly6C^+^ neutrophils initiate a pre-metastatic environment. These TDSF-primed granulocytes travel to the pre-metastatic lung to facilitate angiogenesis, colonization by cancer cells, and subsequent metastasis through activation of one of the Bv8 receptors, prokineticin receptor (PKR)-1 [43]. Downregulation of CTNND1, a member of the subfamily of armadillo (ARM) repeat-containing proteins, increases neutrophil infiltration in the bone and enhances cancer metastasis to the bone in TNBC. Because the knockdown of CTNND1 can upregulate CXCR4 through the PI3K/AKT/HIF-1α pathway, a large number of neutrophils are infiltrated into the bone where CXCL12, the ligand for CXCR4, is high. At the same time, knockdown of CTNND1 can make TNBC cells release a large amount of cytokines such as GM-CSF and IL-8 and promote neutrophils to produce an inhibitory effect on cytotoxic T lymphocytes, which could promote formation of the pre-metastatic niche in the bone [44] (Fig. 4). These factors act in an autocrine or paracrine fashion to render the secondary organ’s microenvironment favorable for colonization of metastatic cancer cells. Specifically, these factors function to (1) induce phenotypic changes in breast cancer cells; (2) recruit bone marrow-derived cells; and (3) form an inflammatory milieu in the microenvironment.

Chemicals such as nicotine can also promote the formation of the pre-metastatic niche through neutrophils in TNBC. Exposure of mice to nicotine leads to pre-metastatic niche formation in the lung by recruiting pro-tumor neutrophils, which induces a mesenchymal-to-epithelial transition (MET) process of breast cancer cells, thus promoting cancer cell colonization and metastasis [45]. Within the pre-metastatic niche, pro-tumor neutrophils inhibit the proliferation and activity of cytotoxic T lymphocytes, promote angiogenesis, and stimulate cancer cell survival, thereby promoting tumor invasion and metastasis. There is mounting evidence that although multiple types of immune cells are involved, neutrophils play a major role in generation of the pre-metastatic niche and, thus, contribute to the metastatic process of TNBC [30, 46].

Neutrophil polarization and NET secretion also contribute to pre-metastatic niche formation. TNBC patients expressing a high level of the nuclear protein high mobility group box 1 protein (HMGB1) are more likely to metastasize. Further investigations revealed that HMGB1 from the primary breast tumor could direct neutrophils to become a NET-producing immunosuppressive type. These data show that CD62L^dim^ neutrophils accelerate lung metastasis by promoting the development of NETs [47].

Through circulation, tumor-derived EVs and TDSFs reach the pre-metastatic site within the target organ. This results in establishment of a cancer-friendly milieu in the target organ prior to cancer cell arrival. In addition, iNOS, PD-L2, and NETs from pro-tumor neutrophils suppress T cells and promote pre-metastatic niche formation in breast cancer.

### Neutrophils as an important prognostic marker of TNBC

An increase in the number of neutrophils is traditionally regarded as an indicator of acute inflammation in pathological conditions. Increasing evidence has revealed the association between neutrophils and cancer prognosis. Patients of different types of cancer frequently show a remarkable increase in the number of circulating neutrophils, often manifested by an increase in the neutrophil-to-lymphocyte ratio (NLR). The NLR is a simple and easily obtainable marker that reflects systemic inflammation and is often used as a prognostic indicator in various types of cancer, including TNBC. Higher NLR values have been associated with poor prognosis, including shorter disease-free survival, overall survival, and higher risk of recurrence in patients with TNBC [48–54].

Except for predicting survival outcomes, changes in NLR are associated with therapeutic response. NLR is associated with a response to neoadjuvant chemotherapy in a cohort consisting of 120 TNBC patients [55]. Furthermore, a high NLR predicts worse disease-free survival in TNBC patients who have failed neoadjuvant chemotherapy or non-metastatic TNBC patients [56–60]. Interestingly, changes in NLR at different time points relative to therapy show different clinical associations, with changes before the start of radiotherapy (odds ratio: 1.115) and one year after surgery (odds ratio: 1.196) associated with increased risk of recurrence or death [61]. Similarly, a cohort study found a strong correlation between the dynamic changes in NLR and disease recurrence as well as the time of death in TNBC patients [62].

In addition to using NLR as a prognostic indicator, the absolute count of neutrophils and the level of neutrophil-derived factors are regarded as being able to predict prognosis in TNBC patients. In single-cell RNA sequencing datasets of TNBC (GSE118389 and GSE75688), higher infiltrating levels of neutrophils and M2 macrophages were reported to be the most efficient signature to predict poor survival compared to other immune cells in TNBC cases [63]. A recent study found that high levels of CXCR2, a chemokine receptor abundantly expressed by neutrophils in TNBC [64], were associated with a subgroup of TNBC patients characterized by better prognosis [65]. CXCL8, a neutrophil-attracting chemokine associated with brain metastasis in TNBC, can be used as a biomarker of poor prognosis of TNBC [66]. Despite these associations, the exact relationship between NLR and breast cancer prognosis is still under investigation and further studies are needed to establish the clinical utility of NLR as a reliable prognostic marker in breast cancer.

### Therapeutic implications

Given their currently known roles in TNBC development, particularly in the metastasizing cascade, neutrophils have been evaluated as a valid target for cancer therapy. In TNBC, approaches targeting neutrophils have shown great promise in slowing down or halting tumor progression in both preclinical (Table 1) and clinical (Table 2) studies [67].

**Table 1:** Preclinical approaches targeting neutrophils in TNBC

Target	Intervention	Effects on neutrophils	References
Arg1/Nos2	l-Arginine supplementation	Reduce effects of arginine depletion	Cao et al. [87]
Cholesterol synthesis	Simvastatin, berberine	Blocking ASPP2-depletion induced NETs formation	Tang et al. [77]
Class I HDACs	Entinostat	Downregulation of Arg1	Christmas et al. [71]
c-MET	OMO-1	Reducing neutrophil infiltration	Steenbrugge et al. [68]
CXCR2	AZD5069	Blocking neutrophil chemotaxis	Safarulla et al. [69]
IL-1R	Anakinra	Reducing propensity for NET formation	Gomes et al. [78]
LXR	RGX-104	Induction of apoptosis	Tavazoie et al. [74]
PDE5	Sildenafil	Downregulation of Arg1 and Nos2	Serafini et al. [70]
RAR/RXR	All-trans retinoic acid	Promoting differentiation of MDSCs into mature cells	Bauer et al. [88]

**Table 2:** Clinical trials investigating neutrophil-targeting agents in TNBC

Target	Intervention	Effects on neutrophils	Status	Clinical trial ID
COX-2	Celebrex	Downregulation of Arg1, Nos2 and COX-2	Completed	NCT00056082
HDACs	Suberoylanilide hydroxamic acid	Inhibiting immunosuppression	Withdrawn	NCT01695057
HDACs	Belinostat	Inhibiting immunosuppression	Recruiting	NCT04315233
HDACs	Vorinostat	Inhibiting immunosuppression	Completed	NCT00574587
HDACs	Entinostat	Inhibiting immunosuppression	Completed	NCT02708680
PTKs	Sorafenib, Apatinib, Famitinib	Induction of apoptosis	Recruiting	NCT05594095
TGFβ	Fresolimumab	Neutralizing TGFβ	Completed	NCT01401062
TGFBR1	Galunisertib	Inhibiting TGFβ receptor I	Active, not recruiting	NCT02672475

Inhibition of chemokine-related pathways, such as CXCR2, leads to the reduction of neutrophil recruitment and, thus, reduces immunosuppression of neutrophils in the immune microenvironment and the circulation [68]. After treatment with CXCR2 inhibitor AZD5069, neutrophil infiltration in brain metastases of TNBC is significantly reduced and, thus, tumor metastasis is significantly inhibited [69].

Some key enzymes of neutrophils, such as Arg1 and Nos2, are directly or indirectly inhibited to reduce their tumor-promoting effects [70, 71]. Interestingly, histone deacetylases (HDACs) are essential enzymes that modify the functions of other enzymes that are important in neutrophils such as Arg1. Therefore, HDAC inhibitors have been reported to increase the efficacy of immune checkpoint inhibition by downregulation of Arg1 in pro-tumor neutrophils [72, 73]. There are several ongoing clinical trials combining HDAC inhibitors and immunotherapy in treating TNBC, and the outcomes from these trials will shed light on the feasibility of this approach.

Promotion of apoptosis of pro-tumor neutrophils or differentiation from pro-tumor into anti-tumor neutrophils to reduce their absolute number weakens the inhibitory effect of neutrophils on other immune cells, thus increasing the sensitivity of breast cancer cells to immunotherapy [74]. In 4T1 tumor bearing mouse models, targeting persistent pro-tumor neutrophils with gemcitabine after primary tumor resection decreases metastatic growth in the lungs [75].

Targeting NETs in breast cancer has also been examined in both preclinical and clinical studies [76–78]. In a mouse model of TNBC metastasis, elimination of the DNA component of the NET using DNase I leads to reduction in the capacity of cancer cells to metastasize [32]. Furthermore, treatment with DNase I or an NE inhibitor (GW311616) significantly decreases the formation of metastases in the liver [30] or in the lung [47] following injection of 4T1 TNBC cells in mice. In the clinical setting, two FDA-approved drugs, aspirin and hydroxychloroquine, have been repurposed as anti-NET treatments. Aspirin, a non-steroidal anti-inflammatory drug, has been shown to halt NET formation and, consequently, patients taking aspirin daily have significantly reduced mortality and decreased risk of distant metastasis of breast cancer [79, 80]. Hydroxychloroquine, an autophagy inhibitor approved to treat many types of cancer [81], has been shown to inhibit the formation of NETs, thus potentiating the responsiveness of TNBC patients to clinical therapy [82].

Apart from these relatively classic targeted treatment methods, a more recent study has reported a new treatment method that targets neutrophils. Linde et al. demonstrate that neutrophils can be harnessed through the combined action of TNF, CD40 agonists, and tumor-binding antibodies to induce tumor eradication and reduce metastasis. It is interesting to note that complements, which are also an important part of the innate immune system, play a key role in this novel mechanism. Complement component C5a activates neutrophils to produce leukotriene B4, further leading to the production of ROS via xanthine oxidase, resulting in oxidative damage. Notably, this mechanism is effective in eliminating multiple tumor types, including TNBC, without requiring the involvement of T cells [83].

## Conclusions

Understanding the immune microenvironment is important for the development of effective immunotherapy strategies for aggressive subtypes of breast cancer. Although much has been learned about the roles of neutrophils in breast cancer, several issues regarding the basic as well as the clinical aspects of the diverse functions of neutrophils in TNBC still exist.

First, the majority of these studies are performed in preclinical models and, thus, limited information is available regarding the roles of neutrophils in the clinical setting [11]. Therefore, studies on neutrophils in TNBC should be performed in more clinically relevant models such as the patient-derived tumor xenograft model and tumor-derived organoids and they should also involve more clinical tissue samples. Some recent studies have examined the involvement of neutrophils in TNBC using patient tissue samples and publicly available databases. Through immunohistochemical and bioinformatics analyses, we found that TNBCs are infiltrated by neutrophils at a significantly higher level and neutrophil-related pathways are indeed dysregulated in TNBC compared with non-TNBC samples. The clinical relevance of these tumor-infiltrating neutrophils needs to be further studied.

Second, neutrophils are fragile cells with a short lifespan (circulatory half-life of 7–10 h in humans) and susceptibility to various treatments. These factors present challenges to the study of neutrophils, particularly in the context of new investigation techniques such as single-cell sequencing. A study of multiple single-cell databases revealed that neutrophils accounted for less than 1.5% of the single-cell sequencing results from various common sequencing platforms. This finding was significantly inconsistent with their percentage in human immune cells. Subsequent analysis identified that neutrophils were difficult to collect using 10× Chromium, the most used single-cell sequencing platform [84]. This may explain why neutrophils have not received much attention in previous single-cell studies. Therefore, improving and developing methods for studying neutrophils represents an urgent need for future investigation.

Third, the identity and roles of distinct forms of neutrophils, e.g., circulating vs. tumor-associated (or tumor-infiltrating), intratumoral vs. stromal, mature vs. immature, should be more clearly defined. How these different forms of neutrophils are related to each other and how they are correlated with clinical outcomes of TNBC patients need to be further examined. Studies should aim at optimizing the specific biomarkers and methods to identify the different forms of neutrophils, particularly those in the different stages of maturation and/or differentiation. From the perspective of pathologists and clinicians, the methods for detecting these biomarkers on neutrophils, e.g., immunohistochemistry together with the specific antibodies used, should be standardized.

Fourth, it has been demonstrated that TNBC can be subdivided into several immune-related subtypes based on the level of immune cell infiltration and the genomic and transcriptomic landscape [85, 86]. How neutrophil alterations correlate with these different immune-related subtypes of TNBC needs to be further studied through basic and translational research on the roles of neutrophils in TNBC and could enable the development of alternative immunotherapeutic strategies for TNBC. These new strategies should aim at targeting neutrophils through either functional or phenotypic manipulation. Specifically, conversion of neutrophils from the pro-tumor toward the anti-tumor phenotype should be examined as a novel approach to the reactivation of anti-tumor immunomodulatory functions of neutrophils. The clinical applications of this special immune cell type as a druggable target can be expected with the hope of benefiting breast cancer patients with better clinical outcomes.

## Data Availability

Not applicable.

## Abbreviations

Arg1: Arginase 1
CAR: Chimeric antigen receptor
CTC: Circulating tumor cell
EMT: Epithelial-to-mesenchymal transition
ER: Estrogen receptor
EV: Extracellular vesicle
G-CSF: Granulocyte colony-stimulating factor
GM-CSF: Granulocyte-monocyte colony-stimulating factor
H_2_O_2_: Hydrogen peroxide
HDAC: Histone deacetylase
HER2: Human epidermal growth factor receptor 2
ICAM1: Intercellular adhesion molecule 1
IFN-β: Interferon-β
IFN-γ: Interferon-γ
iNOS: Inducible nitric oxide synthase
MDSC: Myeloid-derived suppressor cell
MMP9: Matrix metalloproteinase 9
NE: Neutrophil elastase
NET: Neutrophil extracellular trap
NLR: Neutrophil-to-lymphocyte ratio
NO: Nitric oxide
O_2_‧: Superoxide anion
PR: Progesterone receptor
PROK2: Prokineticin 2
RNS: Reactive nitrogen species
ROS: Reactive oxygen species
TAM: Tumor-associated macrophage
TAN: Tumor-associated neutrophil
TDSF: Tumor-derived secreted factor
TGF-β: Transforming growth factor-β
TIMP1: Tissue inhibitor of metalloproteinase 1
TIN: Tumor-infiltrating neutrophil
TME: Tumor microenvironment
TNBC: Triple-negative breast cancer
VEGF: Vascular endothelial growth factor

## References

[1] Lecot P, Sarabi M, Pereira Abrantes M, Mussard J, Koenderman L, Caux C, Bendriss-Vermare N, Michallet MC (2019). Neutrophil heterogeneity in cancer: from biology to therapies. Front Immunol.

[2] Liew PX, Kubes P (2019). The neutrophil's role during health and disease. Physiol Rev.

[3] Quail DF, Amulic B, Aziz M, Barnes BJ, Eruslanov E, Fridlender ZG, Goodridge HS, Granot Z, Hidalgo A, Huttenlocher A (2022). Neutrophil phenotypes and functions in cancer: a consensus statement. J Exp Med.

[4] Hajizadeh F, Aghebati Maleki L, Alexander M, Mikhailova MV, Masjedi A, Ahmadpour M, Hashemi V, Jadidi-Niaragh F (2021). Tumor-associated neutrophils as new players in immunosuppressive process of the tumor microenvironment in breast cancer. Life Sci.

[5] Mouchemore KA, Anderson RL, Hamilton JA (2018). Neutrophils, G-CSF and their contribution to breast cancer metastasis. FEBS J.

[6] Hedrick CC, Malanchi I (2022). Neutrophils in cancer: heterogeneous and multifaceted. Nat Rev Immunol.

[7] Sung H, Ferlay J, Siegel RL, Laversanne M, Soerjomataram I, Jemal A, Bray F (2021). Global Cancer Statistics 2020: GLOBOCAN Estimates of Incidence and Mortality Worldwide for 36 Cancers in 185 Countries. CA Cancer J Clin.

[8] Bianchini G, De Angelis C, Licata L, Gianni L (2022). Treatment landscape of triple-negative breast cancer—Expanded options, evolving needs. Nat Rev Clin Oncol.

[9] Foulkes WD, Smith IE, Reis-Filho JS (2010). Triple-negative breast cancer. N Engl J Med.

[10] Soto-Perez-de-Celis E, Chavarri-Guerra Y, Leon-Rodriguez E, Gamboa-Dominguez A (2017). Tumor-associated neutrophils in breast cancer subtypes. Asian Pac J Cancer Prev.

[11] Coffelt SB, Wellenstein MD, de Visser KE (2016). Neutrophils in cancer: neutral no more. Nat Rev Cancer.

[12] SenGupta S, Subramanian BC, Parent CA (2019). Getting TANned: How the tumor microenvironment drives neutrophil recruitment. J Leukoc Biol.

[13] Yu M, Stott S, Toner M, Maheswaran S, Haber DA (2011). Circulating tumor cells: approaches to isolation and characterization. J Cell Biol.

[14] Pereira-Veiga T, Schneegans S, Pantel K, Wikman H (2022). Circulating tumor cell-blood cell crosstalk: Biology and clinical relevance. Cell Rep.

[15] Aceto N, Bardia A, Miyamoto DT, Donaldson MC, Wittner BS, Spencer JA, Yu M, Pely A, Engstrom A, Zhu H (2014). Circulating tumor cell clusters are oligoclonal precursors of breast cancer metastasis. Cell.

[16] Szczerba BM, Castro-Giner F, Vetter M, Krol I, Gkountela S, Landin J, Scheidmann MC, Donato C, Scherrer R, Singer J (2019). Neutrophils escort circulating tumour cells to enable cell cycle progression. Nature.

[17] Casbon AJ, Reynaud D, Park C, Khuc E, Gan DD, Schepers K, Passegue E, Werb Z (2015). Invasive breast cancer reprograms early myeloid differentiation in the bone marrow to generate immunosuppressive neutrophils. Proc Natl Acad Sci USA.

[18] Jenne CN, Kubes P (2016). Gastrointestinal cancer: neutrophils and cancer: guilt by association. Nat Rev Gastroenterol Hepatol.

[19] Wang Y, Chen J, Yang L, Li J, Wu W, Huang M, Lin L, Su S (2019). Tumor-contacted neutrophils promote metastasis by a CD90-TIMP-1 Juxtacrine-paracrine loop. Clin Cancer Res.

[20] Fridlender ZG, Sun J, Kim S, Kapoor V, Cheng G, Ling L, Worthen GS, Albelda SM (2009). Polarization of tumor-associated neutrophil phenotype by TGF-beta: "N1" versus "N2" TAN. Cancer Cell.

[21] Li YC, Zou JM, Luo C, Shu Y, Luo J, Qin J, Wang Y, Li D, Wang SS, Chi G (2017). Circulating tumor cells promote the metastatic colonization of disseminated carcinoma cells by inducing systemic inflammation. Oncotarget.

[22] Andzinski L, Kasnitz N, Stahnke S, Wu CF, Gereke M, von Kockritz-Blickwede M, Schilling B, Brandau S, Weiss S, Jablonska J (2016). Type I IFNs induce anti-tumor polarization of tumor associated neutrophils in mice and human. Int J Cancer.

[23] SenGupta S, Hein LE, Xu Y, Zhang J, Konwerski JR, Li Y, Johnson C, Cai D, Smith JL, Parent CA (2021). Triple-negative breast cancer cells recruit neutrophils by secreting TGF-β and CXCR2 ligands. Front Immunol.

[24] Tokumaru Y, Oshi M, Murthy V, Tian W, Yan L, Angarita FA, Nagahashi M, Matsuhashi N, Futamura M, Yoshida K (2021). Low intratumoral genetic neutrophil-to-lymphocyte ratio (NLR) is associated with favorable tumor immune microenvironment and with survival in triple negative breast cancer (TNBC). Am J Cancer Res.

[25] Singhal S, Bhojnagarwala PS, O'Brien S, Moon EK, Garfall AL, Rao AS, Quatromoni JG, Stephen TL, Litzky L, Deshpande C (2016). Origin and role of a subset of tumor-associated neutrophils with antigen-presenting cell features in early-stage human lung cancer. Cancer Cell.

[26] Houghton AM, Rzymkiewicz DM, Ji H, Gregory AD, Egea EE, Metz HE, Stolz DB, Land SR, Marconcini LA, Kliment CR (2010). Neutrophil elastase-mediated degradation of IRS-1 accelerates lung tumor growth. Nat Med.

[27] Jablonska J, Lang S, Sionov RV, Granot Z (2017). The regulation of pre-metastatic niche formation by neutrophils. Oncotarget.

[28] Branzk N, Papayannopoulos V (2013). Molecular mechanisms regulating NETosis in infection and disease. Semin Immunopathol.

[29] Shao BZ, Yao Y, Li JP, Chai NL, Linghu EQ (2021). The role of neutrophil extracellular traps in cancer. Front Oncol.

[30] Hsu BE, Tabaries S, Johnson RM, Andrzejewski S, Senecal J, Lehuede C, Annis MG, Ma EH, Vols S, Ramsay L (2019). Immature low-density neutrophils exhibit metabolic flexibility that facilitates breast cancer liver metastasis. Cell Rep.

[31] Park J, Wysocki RW, Amoozgar Z, Maiorino L, Fein MR, Jorns J, Schott AF, Kinugasa-Katayama Y, Lee Y, Won NH (2016). Cancer cells induce metastasis-supporting neutrophil extracellular DNA traps. Sci Transl Med.

[32] Leal AC, Mizurini DM, Gomes T, Rochael NC, Saraiva EM, Dias MS, Werneck CC, Sielski MS, Vicente CP, Monteiro RQ (2017). Tumor-derived exosomes induce the formation of neutrophil extracellular traps: implications for the establishment of cancer-associated thrombosis. Sci Rep.

[33] Cedervall J, Zhang Y, Huang H, Zhang L, Femel J, Dimberg A, Olsson AK (2015). Neutrophil extracellular traps accumulate in peripheral blood vessels and compromise organ function in tumor-bearing animals. Cancer Res.

[34] Albrengues J, Shields MA, Ng D, Park CG, Ambrico A, Poindexter ME, Upadhyay P, Uyeminami DL, Pommier A, Kuttner V (2018). Neutrophil extracellular traps produced during inflammation awaken dormant cancer cells in mice. Science.

[35] Kaplan RN, Riba RD, Zacharoulis S, Bramley AH, Vincent L, Costa C, MacDonald DD, Jin DK, Shido K, Kerns SA (2005). VEGFR1-positive haematopoietic bone marrow progenitors initiate the pre-metastatic niche. Nature.

[36] Psaila B, Lyden D (2009). The metastatic niche: adapting the foreign soil. Nat Rev Cancer.

[37] Liu Y, Cao X (2016). Characteristics and significance of the pre-metastatic niche. Cancer Cell.

[38] Dong Q, Liu X, Cheng K, Sheng J, Kong J, Liu T (2021). Pre-metastatic niche formation in different organs induced by tumor extracellular vesicles. Front Cell Dev Biol.

[39] Yang X, Zhang Y, Zhang Y, Zhang S, Qiu L, Zhuang Z, Wei M, Deng X, Wang Z, Han J (2021). The key role of exosomes on the pre-metastatic niche formation in tumors. Front Mol Biosci.

[40] Gu P, Sun M, Li L, Yang Y, Jiang Z, Ge Y, Wang W, Mu W, Wang H (2021). Breast tumor-derived exosomal MicroRNA-200b-3p promotes specific organ metastasis through regulating ccl2 expression in lung epithelial cells. Front Cell Dev Biol.

[41] Qi M, Xia Y, Wu Y, Zhang Z, Wang X, Lu L, Dai C, Song Y, Xu K, Ji W (2022). Lin28B-high breast cancer cells promote immune suppression in the lung pre-metastatic niche via exosomes and support cancer progression. Nat Commun.

[42] Li R, Wen A, Lin J (2020). Pro-inflammatory cytokines in the formation of the pre-metastatic niche. Cancers (Basel).

[43] Kowanetz M, Wu X, Lee J, Tan M, Hagenbeek T, Qu X, Yu L, Ross J, Korsisaari N, Cao T (2010). Granulocyte-colony stimulating factor promotes lung metastasis through mobilization of Ly6G+Ly6C+ granulocytes. Proc Natl Acad Sci USA.

[44] Lin Q, Fang X, Liang G, Luo Q, Cen Y, Shi Y, Jia S, Li J, Yang W, Sanders AJ (2021). Silencing CTNND1 mediates triple-negative breast cancer bone metastasis via upregulating CXCR4/CXCL12 axis and neutrophils infiltration in bone. Cancers (Basel).

[45] Tyagi A, Sharma S, Wu K, Wu SY, Xing F, Liu Y, Zhao D, Deshpande RP, D'Agostino RB, Watabe K (2021). Nicotine promotes breast cancer metastasis by stimulating N2 neutrophils and generating pre-metastatic niche in lung. Nat Commun.

[46] Tabaries S, Ouellet V, Hsu BE, Annis MG, Rose AA, Meunier L, Carmona E, Tam CE, Mes-Masson AM, Siegel PM (2015). Granulocytic immune infiltrates are essential for the efficient formation of breast cancer liver metastases. Breast Cancer Res.

[47] Wang Z, Yang C, Li L, Jin X, Zhang Z, Zheng H, Pan J, Shi L, Jiang Z, Su K (2020). Tumor-derived HMGB1 induces CD62L(dim) neutrophil polarization and promotes lung metastasis in triple-negative breast cancer. Oncogenesis.

[48] Ethier JL, Desautels D, Templeton A, Shah PS, Amir E (2017). Prognostic role of neutrophil-to-lymphocyte ratio in breast cancer: a systematic review and meta-analysis. Breast Cancer Res.

[49] Orditura M, Galizia G, Diana A, Saccone C, Cobellis L, Ventriglia J, Iovino F, Romano C, Morgillo F, Mosca L (2016). Neutrophil to lymphocyte ratio (NLR) for prediction of distant metastasis-free survival (DMFS) in early breast cancer: a propensity score-matched analysis. ESMO Open.

[50] Kim KM, Choi HS, Noh H, Cho IJ, Lim ST, Lee JI, Han A (2021). Neutrophil to lymphocyte ratio after treatment completion as a potential predictor of survival in patients with triple-negative breast cancer. J Breast Cancer.

[51] Pang J, Zhou H, Dong X, Wang S, Xiao Z (2021). Relationship between the neutrophil to lymphocyte ratio, stromal tumor-infiltrating lymphocytes, and the prognosis and response to neoadjuvant chemotherapy in triple-negative breast cancer. Clin Breast Cancer.

[52] Patel DA, Xi J, Luo J, Hassan B, Thomas S, Ma CX, Campian JL (2019). Neutrophil-to-lymphocyte ratio as a predictor of survival in patients with triple-negative breast cancer. Breast Cancer Res Treat.

[53] Polley MC, Leon-Ferre RA, Leung S, Cheng A, Gao D, Sinnwell J, Liu H, Hillman DW, Eyman-Casey A, Gilbert JA (2021). A clinical calculator to predict disease outcomes in women with triple-negative breast cancer. Breast Cancer Res Treat.

[54] Zenan H, Zixiong L, Zhicheng Y, Mei H, Xiongbin Y, Tiantian W, Min D, Renbin L, Changchang J (2019). Clinical prognostic evaluation of immunocytes in different molecular subtypes of breast cancer. J Cell Physiol.

[55] Lusho S, Durando X, Mouret-Reynier MA, Kossai M, Lacrampe N, Molnar I, Penault-Llorca F, Radosevic-Robin N, Abrial C (2021). Platelet-to-lymphocyte ratio is associated with favorable response to neoadjuvant chemotherapy in triple negative breast cancer: a study on 120 patients. Front Oncol.

[56] Dong X, Liu C, Yuan J, Wang S, Ding N, Li Y, Wu Y, Xiao Z (2021). Prognostic roles of neutrophil-to-lymphocyte ratio and stromal tumor-infiltrating lymphocytes and their relationship in locally advanced triple-negative breast cancer treated with neoadjuvant chemotherapy. Breast Care (Basel).

[57] Chae S, Kang KM, Kim HJ, Kang E, Park SY, Kim JH, Kim SH, Kim SW, Kim EK (2018). Neutrophil-lymphocyte ratio predicts response to chemotherapy in triple-negative breast cancer. Curr Oncol.

[58] Shi K, Westhuyzen J, Gortman A, Shakespeare TP, Aherne NJ (2022). Prognostic value of the neutrophil-lymphocyte ratio in triple negative breast cancer patients. Ann Clin Lab Sci.

[59] Lou C, Jin F, Zhao Q, Qi H (2022). Correlation of serum NLR, PLR and HALP with efficacy of neoadjuvant chemotherapy and prognosis of triple-negative breast cancer. Am J Transl Res.

[60] Qiu X, Song Y, Cui Y, Liu Y (2018). Increased neutrophil-lymphocyte ratio independently predicts poor survival in non-metastatic triple-negative breast cancer patients. IUBMB Life.

[61] Kim JH, Son NH, Lee JS, Mun JE, Kim JY, Park HS, Park S, Kim SI, Park BW (2021). Time-sequencing of the neutrophil-to-lymphocyte ratio to predict prognosis of triple-negative breast cancer. Cancers (Basel).

[62] Moldoveanu D, Pravongviengkham V, Best G, Martinez C, Hijal T, Meguerditchian AN, Lajoie M, Dumitra S, Watson I, Meterissian S (2020). Dynamic neutrophil-to-lymphocyte ratio: a novel prognosis measure for triple-negative breast cancer. Ann Surg Oncol.

[63] Jiang K, Dong M, Li C, Sheng J (2021). Unraveling heterogeneity of tumor cells and microenvironment and its clinical implications for triple negative breast cancer. Front Oncol.

[64] Boissiere-Michot F, Jacot W, Fraisse J, Gourgou S, Timaxian C, Lazennec G (2020). Prognostic value of CXCR2 in breast cancer. Cancers (Basel).

[65] Boissiere-Michot F, Jacot W, Massol O, Mollevi C, Lazennec G (2021). CXCR2 levels correlate with immune infiltration and a better prognosis of triple-negative breast cancers. Cancers (Basel).

[66] Shen Y, Zhang B, Wei X, Guan X, Zhang W (2022). CXCL8 is a prognostic biomarker and correlated with TNBC brain metastasis and immune infiltration. Int Immunopharmacol.

[67] Zhao Y, Rahmy S, Liu Z, Zhang C, Lu X (2020). Rational targeting of immunosuppressive neutrophils in cancer. Pharmacol Ther.

[68] Steenbrugge J, Vander Elst N, Demeyere K, De Wever O, Sanders NN, Van Den Broeck W, Ciamporcero E, Perera T, Meyer E (2021). OMO-1 reduces progression and enhances cisplatin efficacy in a 4T1-based non-c-MET addicted intraductal mouse model for triple-negative breast cancer. NPJ Breast Cancer.

[69] Safarulla S, Madan A, Xing F, Chandrasekaran A (2022). CXCR2 mediates distinct neutrophil behavior in brain metastatic breast tumor. Cancers (Basel).

[70] Serafini P, Meckel K, Kelso M, Noonan K, Califano J, Koch W, Dolcetti L, Bronte V, Borrello I (2006). Phosphodiesterase-5 inhibition augments endogenous antitumor immunity by reducing myeloid-derived suppressor cell function. J Exp Med.

[71] Christmas BJ, Rafie CI, Hopkins AC, Scott BA, Ma HS, Cruz KA, Woolman S, Armstrong TD, Connolly RM, Azad NA (2018). Entinostat converts immune-resistant breast and pancreatic cancers into checkpoint-responsive tumors by reprogramming tumor-infiltrating MDSCs. Cancer Immunol Res.

[72] Kim K, Skora AD, Li Z, Liu Q, Tam AJ, Blosser RL, Diaz LA, Papadopoulos N, Kinzler KW, Vogelstein B (2014). Eradication of metastatic mouse cancers resistant to immune checkpoint blockade by suppression of myeloid-derived cells. Proc Natl Acad Sci USA.

[73] Orillion A, Hashimoto A, Damayanti N, Shen L, Adelaiye-Ogala R, Arisa S, Chintala S, Ordentlich P, Kao C, Elzey B (2017). Entinostat neutralizes myeloid-derived suppressor cells and enhances the antitumor effect of PD-1 inhibition in murine models of lung and renal cell carcinoma. Clin Cancer Res.

[74] Tavazoie MF, Pollack I, Tanqueco R, Ostendorf BN, Reis BS, Gonsalves FC, Kurth I, Andreu-Agullo C, Derbyshire ML, Posada J (2018). LXR/ApoE activation restricts innate immune suppression in cancer. Cell.

[75] Bosiljcic M, Cederberg RA, Hamilton MJ, LePard NE, Harbourne BT, Collier JL, Halvorsen EC, Shi R, Franks SE, Kim AY (2019). Targeting myeloid-derived suppressor cells in combination with primary mammary tumor resection reduces metastatic growth in the lungs. Breast Cancer Res.

[76] Snoderly HT, Boone BA, Bennewitz MF (2019). Neutrophil extracellular traps in breast cancer and beyond: current perspectives on NET stimuli, thrombosis and metastasis, and clinical utility for diagnosis and treatment. Breast Cancer Res.

[77] Tang Q, Liang B, Zhang L, Li X, Li H, Jing W, Jiang Y, Zhou F, Zhang J, Meng Y (2022). Enhanced CHOLESTEROL biosynthesis promotes breast cancer metastasis via modulating CCDC25 expression and neutrophil extracellular traps formation. Sci Rep.

[78] Gomes T, Varady CBS, Lourenco AL, Mizurini DM, Rondon AMR, Leal AC, Goncalves BS, Bou-Habib DC, Medei E, Monteiro RQ (2019). IL-1beta blockade attenuates thrombosis in a neutrophil extracellular trap-dependent breast cancer model. Front Immunol.

[79] Holmes MD, Chen WY, Li L, Hertzmark E, Spiegelman D, Hankinson SE (2010). Aspirin intake and survival after breast cancer. J Clin Oncol.

[80] Rothwell PM, Wilson M, Price JF, Belch JF, Meade TW, Mehta Z (2012). Effect of daily aspirin on risk of cancer metastasis: a study of incident cancers during randomised controlled trials. Lancet.

[81] Mohsen S, Sobash PT, Algwaiz GF, Nasef N, Al-Zeidaneen SA, Karim NA (2022). Autophagy agents in clinical trials for cancer therapy: a brief review. Curr Oncol.

[82] Cook KL, Warri A, Soto-Pantoja DR, Clarke PA, Cruz MI, Zwart A, Clarke R (2014). Hydroxychloroquine inhibits autophagy to potentiate antiestrogen responsiveness in ER+ breast cancer. Clin Cancer Res.

[83] Linde IL, Prestwood TR, Qiu J, Pilarowski G, Linde MH, Zhang X, Shen L, Reticker-Flynn NE, Chiu DK, Sheu LY (2023). Neutrophil-activating therapy for the treatment of cancer. Cancer Cell.

[84] Salcher S, Sturm G, Horvath L, Untergasser G, Kuempers C, Fotakis G, Panizzolo E, Martowicz A, Trebo M, Pall G (2022). High-resolution single-cell atlas reveals diversity and plasticity of tissue-resident neutrophils in non-small cell lung cancer. Cancer Cell.

[85] Jiang YZ, Ma D, Suo C, Shi J, Xue M, Hu X, Xiao Y, Yu KD, Liu YR, Yu Y (2019). Genomic and transcriptomic landscape of triple-negative breast cancers: subtypes and treatment strategies. Cancer Cell.

[86] Xiao Y, Ma D, Zhao S, Suo C, Shi J, Xue MZ, Ruan M, Wang H, Zhao J, Li Q (2019). Multi-s. Clin Cancer Res.

[87] Cao Y, Feng Y, Zhang Y, Zhu X, Jin F (2016). L-Arginine supplementation inhibits the growth of breast cancer by enhancing innate and adaptive immune responses mediated by suppression of MDSCs in vivo. BMC Cancer.

[88] Bauer R, Udonta F, Wroblewski M, Ben-Batalla I, Santos IM, Taverna F, Kuhlencord M, Gensch V, Pasler S, Vinckier S (2018). Blockade of myeloid-derived suppressor cell expansion with all-trans retinoic acid increases the efficacy of antiangiogenic therapy. Cancer Res.

